# Blocking Nuclear Factor-Kappa B Protects against Diet-Induced Hepatic Steatosis and Insulin Resistance in Mice

**DOI:** 10.1371/journal.pone.0149677

**Published:** 2016-03-01

**Authors:** Tianshu Zeng, Jing Zhou, Linzheng He, Juan Zheng, Lulu Chen, Chaodong Wu, Wenfang Xia

**Affiliations:** 1 Department of Endocrinology, Union Hospital, Tongji Medical College, Huazhong University of Science and Technology, Wuhan, 430022, China; 2 Department of Endocrinology, Chengdu First People’s Hospital, Chengdu, 610000, China; 3 Department of Nutrition and Food Science, Texas A&M University, College Station, Texas, 77843, United States of America; Bambino Gesù Children's Hospital, ITALY

## Abstract

Inflammation critically contributes to the development of various metabolic diseases. However, the effects of inhibiting inflammatory signaling on hepatic steatosis and insulin resistance, as well as the underlying mechanisms remain obscure. In the current study, male C57BL/6J mice were fed a chow diet or high-fat diet (HFD) for 8 weeks. HFD-fed mice were respectively treated with p65 siRNA, non-silence control siRNA or vehicle every 4^th^ day for the last 4 weeks. Vehicle-treated (HF) and non-silence siRNA-treated (HFNS) mice displayed overt inflammation, hepatic steatosis and insulin resistance compared with chow-diet-fed (NC) mice. Upon treatment with NF-κB p65 siRNA, HFD-fed (HFPS) mice were protected from hepatic steatosis and insulin resistance. Furthermore, Atg7 and Beclin1 expressions and p-AMPK were increased while p-mTOR was decreased in livers of HFPS mice in relative to HF and HFNS mice. These results suggest a crosslink between NF-κB signaling pathway and liver AMPK/mTOR/autophagy axis in the context of hepatic steatosis and insulin resistance.

## Introduction

Insulin resistance is a hallmark of Type-2 diabetes and evidence demonstrates chronic low-grade inflammation as a contributor to the initiation and perpetuation of insulin resistance [1, 2]. Plenty of proinflammatory factors are involved in such low-grade inflammation [3]. The mechanisms responsible for these inflammation-induced deleterious changes provide considerable therapeutic targets for drug discovery. High doses of sodium salicylate were observed to attenuate glycosuria more than a century ago [2]. Nowadays several studies have provided clues that nuclear factor kappa B (NF-κB) may be a principal regulator of pathways downstream of calorie excess that produce detrimental effects on glucose homeostasis and insulin sensitivity[4–6] and, insulin resistance is partially promoted by a shift of macrophage polarization from alterative M2 activation state to classic M1 activation state, during which process, activated M1 macrophages non-specific markers *F4/80*, *CD68* and specific marker *CD11c* were upregulated, driven by NF-κB signaling [7]. Furthermore, selective inhibition of NF-κB in hypothalamus has been found to protect experimental animals from diet-related insulin resistance [8].

Accumulating evidence also suggests that autophagy, an evolutionarily conserved programmed process, impacts diverse cellular processes and plays a crucial role in lipid and glycogen metabolism, and the regulation of inflammation responses [9, 10]. Autophagy-related genes like *Atg7* and *Beclin1* encode for protein products, which are essential components of the functional complex mediating autophagic process. Defects in autophagy are observed in various pathological conditions including obesity, steatosis, cancer and neurodegenerative diseases [11–13]. Of note, autophagy defect in skeletal muscle can induce NF-κB -mediated inflammation [14]. Restoring autophagic flux in liver has been proved to suppress inflammation and ameliorate insulin resistance by Park et al.[15] very recently. The expressions of genes involved in lipogenesis (acetyl CoA carboxylase (*ACC*), fatty acid synthase (*FAS*), carnitine palmitoyltransferase 1A (*CPT1A*) and sterol regulatory element binding protein-1c (*SREBP1c*)) and in fatty acid oxidation (acyl-CoA oxidase 1 (*Acox1*)) were altered according to various nutrition conditions in vivo and in vitro, ultimately leading to lipid accumulation or clearance in tissues via depressed or enhanced autophagy. Intracellular lipid content, hepatic lipid deposition and insulin resistance after lipid loading are increased as a result of autophagy insufficiency [16]. These hint at the exciting possibility that targeting the diet-induced branch of NF-κB inflammation signaling somehow correlated with remodeling autophagy could have beneficial effects on improving insulin resistance in type-2 diabetes without affecting pivotal immune functions.

Small interfering RNA (siRNA) has been widely applied in biomedicine because of its specific and efficient gene silencing. Data from preclinical programs suggest that siRNA therapeutics have the potency for treating diseases. And many clinical trials of siRNA-based therapeutics have been carried out [17]. Due to its strong transcriptional activity, the p65 subunit of NF-κB is responsible for most of NF-κB’s transcriptional activity. Here, we utilized chemically modified P65 siRNA to explore the pathophysiological connections between insulin resistance, NF-κB inflammatory pathways and liver autophagy in HFD feeding mice and to reveal the molecular mechanisms underlying the alteration of autophagy responses as well as their impacts on insulin signaling in target tissues.

## Materials and Methods

### P65 siRNA preparation

P65 siRNA and non-silence control siRNA (NC siRNA) were purchased from Guangzhou RiboBio Co., Ltd. (Guangdong, China). P65 siRNA sense sequence is 5’- CAAGATCAATGGCTACACAdTdT-3’ and the antisense sequence is 3’-dTdT GUUCUAGUUACCGAUGUGU-5’. The NC siRNA sense sequence is 5'-GGCCUCAGCUGCGCGACGCdTdT-3', and the antisense sequence is 5'-GCGUCGCGCAGCUGGGCCAdTdT-3'. Both siRNA were modified by 5’ Cholesterol and 2’OMe to prolong each half life to about 96h [18].

### Animal experiments

Six-week-old C57 BL/6j mice, purchased from Wuhan University Center for Animal Experiment/A3-Lab, were housed individually in an environmentally controlled room (18°C–21°C, 40%–70% relative humidity) with ad libitum access to water and food and kept under a 12-hour dark/12-hour light cycle. After 4 weeks of acclimatization reared with chow diet, normal control mice (NC, n = 10) were continuously fed a chow diet, whereas high fat diet mice, classified as high-fat diet group (HF, n = 10), high-fat diet with non-silence siRNA group (HFNS, n = 10) and high-fat diet with P65 siRNA group (HFPS, n = 10), received a diet (D12492, Research Diets, Beijing HFK Bioscience Co., Ltd., China) with 60% kcal% fat for 8 weeks. During the last 4 weeks of dietary treatment, mice of HFPS and HFNS received a tail vein injection of P65 siRNA and negative control (NC siRNA) dissolved in 0.2 ml normal saline respectively, and an equal amount of normal saline alone for the other two groups every 4^th^ day. The siRNAs were injected 15nmol (about 8 mg/Kg body weight) per mouse every time. The treatment was well-tolerated and there were no dropouts. At the end of experiments, mice were killed by cervical dislocation after a 6 h period of fasting; blood samples and tissues were harvested and stored at -80°C for further analysis. The Institutional Animal Care and Use Committee at the Tongji Medical College, Huazhong University of Science and Technology approved all animal procedures.(license no. SYXK 2010–0057)

### Metabolic Phenotyping

Mouse body weight was regularly measured. Serum insulin levels were measured at certain intervals throughout the animal experiments using commercially available ELISA kits (ALPCO Diagnostics, Windham, NH), during which detection blood samples were collected from the orbital sinus of mice. After acclimatization, at the end of 4th and 8th week, overnight-fasted or 6h-fasted mice were intraperitoneally injected with glucose (2g/kg of body weight) or insulin (1U/kg of body weight, Novolin R; Novo Nordisk, Bagsværd, Denmark) for the glucose and insulin tolerance test respectively; blood glucose concentrations were measured at the indicated time points post-injection from tail blood with the One Touch blood glucose monitoring system (LifeScan Inc., Milpitas, CA). Homeostasis model assessment index-insulin resistance (HOMA-IR) was calculated as follows: HOMA-IR = fasting serum insulin (mU/L) × fasting plasma glucose (mM)/22.5 [19]. Serum and liver triglyceride (TG) and serum total cholesterol (TC) were determined with triglyceride and total cholesterol test kits (Nanjing Jian Cheng Bioengineering Institute, Jiangsu, China) according to the manufacturers’ instructions. The levels of fatty acid in blood and tissues (liver, epididymal fat, subcutaneous fat and gastrocnemius) were quantified using specific ELISA kits from Nanjing Jian Cheng.

### Tissue histology

Liver tissues were fixed in buffered 4% paraformaldehyde. Then fixed tissues were dehydrated by graded ethanol and xylene and embedded in paraffin. Sections (4 μm thick) were stained with H&E method. All the images were visualized and captured with a Motic microscope BA310 (Ted Pella, Inc., Los Angeles, CA) equipped with a digital camera. Image-Pro Plus software was then used to determine the degree of lipid droplets of the immunohistochemical images.

### Cytokines and NF-κB activation assay

The concentrations of tumor necrosis factor-a (TNF-a) and interleukin-6 (IL-6) in the serum and liver, epididymal fat, subcutaneous fat and gastrocnemius tissues as well as p65 activity (i.e. NF-κB DNA binding activity) in liver and epididymal fat were measured using commercially available ELISA kits (RayBiotech, Inc., Norcross, GA and Cayman chemical company, Ann Arbor, MI respectively) according to the manufacturers’ protocols. The plates were read on an EL800 ELISA plate reader (BioTek Instruments Ltd., Potton, UK). All samples were run in duplicates and analyzed on the same day to eliminate day-to-day variation.

### Western blot analyses

Mice were intraperitoneally injected with saline or Novolin R (1.5 U/Kg body weight) 0.5h before killed. Liver and gastrocnemius tissues lysis, protein extraction, and western blot analyses were performed as described previously [20]. Briefly, tissues were dissolved in a lysis buffer (Beyotime, Shanghai, China) containing protease inhibitors and phosphatase inhibitor according to the manufacturer’s protocol. Equal amounts of protein extracts were separated by SDS/PAGE, then transferred to a PVDF membrane (Millipore, Boston, MA), followed by blocking for 1.5 hours at room temperature, incubating with primary antibody in different recommended dilutions at 4°C overnight and horseradish peroxidase-conjugated secondary antibody for 1 hour at room temperature. Primary antibodies included rabbit anti-Akt, anti- phosphorylated Akt (Ser473), anti-AMPKα, anti- phosphorylated AMPKα (Thr172), anti-mTOR, anti-phosphorylated mTOR (Ser2448) and mouse anti-GAPDH. Secondary antibody were HRP-conjugatedanti-rabbit or -mouse IgGs. The blots were detected using enhanced chemiluminescence detection kit (Beyotime, Shanghai, China) and densitometric analyses of Western blotting images were performed using Image J software (National Institutes of Health). Antibodies were purchased from Cell Signaling Tech. (Beverly, MA, USA) except for GAPDH monoclonal mouse mAb (Abbkine, USA).

### RT-qPCR

Total RNAs from liver tissues were extracted using TRIzol (Qiagen, Valencia, CA). Complementary DNA was synthesized using the Prime Script RT reagent kit (Takara Biotechnology Co., Ltd., Japan) according to the manufacturer’s protocol. Then PCR was performed and quantified using SYBR Green real-time PCR Master Mix (Takara Biotechnology Co., Ltd., Japan) at a LightCycler480-PCR machine (Roche Diagnostics, Mannheim, Germany). Gene expression levels were calculated after normalization to the standard housekeeping genes GAPDH, using the comparative CT method as described previously [21]. Briefly, the gene expression levels were quantified as follows: Fold change = 2^−ΔΔCt^ = 2^- [(Ct gene of interest—Ct GAPDH) sample A—(Ct gene of interest—Ct GAPDH)sample B]^. The primers were synthesized and purchased from Shangon Biotech (Shanghai, China), and the sequences are presented in Table 1.

**Table 1:** Primer sequences for qPCR analysis.

Target	Direction	Sequence
*GAPDH*	Sense	5’ GGTGAAGGTCGGTGTGAACG 3’
	Anti-sense	5’ CTCGCTCCTGGAAGATGGTG 3’
*CD68*	Sense	5’ CTTCGGGCCATGTTTCTCTT 3’
	Anti-sense	5’ ATTGTCGTCTGCGGGTGAT 3’
*F4/80*	Sense	5’ GCTGTGAGATTGTGGAAGCA 3’
	Anti-sense	5’ GGCAAGACATACCAGGGAGA 3’
*CD11c*	Sense	5’ GGTGAAGGTCGGTGTGAACG 3’
	Anti-sense	5’ CATCAGGGAGAACCGTGTG 3’
*Atg7*	Sense	5’ATGCCAGGACACCCTGTGAACTTC3’
	Anti-sense	5’ACATCATTGCAGAAGTAGCAGCCA3’
*Beclin1*	Sense	5’ AGCCTCTGAAACTGGACACG3’
	Anti-sense	5’ TAGCCTCTTCCTCCTGGGTCT3’
*ACC*	Sense	5’TTTCTTCCTTCGCCTCCTTT3’
	Anti-sense	5’GCCAATCTCATTTCCTCCT3’
*FAS*	Sense	5’AAATTCAGCCCGTTGGAGT3’
	Anti-sense	5’AAGTTGCATCCACCCAAATC3’
*CPT1A*	Sense	5’TCAAGCCAGACGAAGAACATC3’
	Anti-sense	5’TGGTAGGAGAGCAGCACCTT3’
*SREBP1c*	Sense	5’GAGGCAGAGAGCAGAGATGG3’
	Anti-sense	5’GACAAAGAGAAGAGCCAAGCA3’
*Acox1*	Sense	5’ATCAAGAGAAGCGAGCCAGA3’
	Anti-sense	5’CCGAGAAAGTGGAAGGCATA3’

### Statistical analysis

Results are presented as mean ± standard error (SE). The data was, or can be converted to be normally distributed. Statistical analyses were performed to assess the differences among the groups at the same sampling points using one-way analysis of variance followed by the Bonferroni post-hoc test for multiple comparisons with GraphPad Prism 5.0 (GraphPad Software Inc., San Diego, CA, USA). p<0.05 was considered statistically significant.

## Results

### P65 siRNA regulated HFD-induced inflammatory response

**Fig 1:**
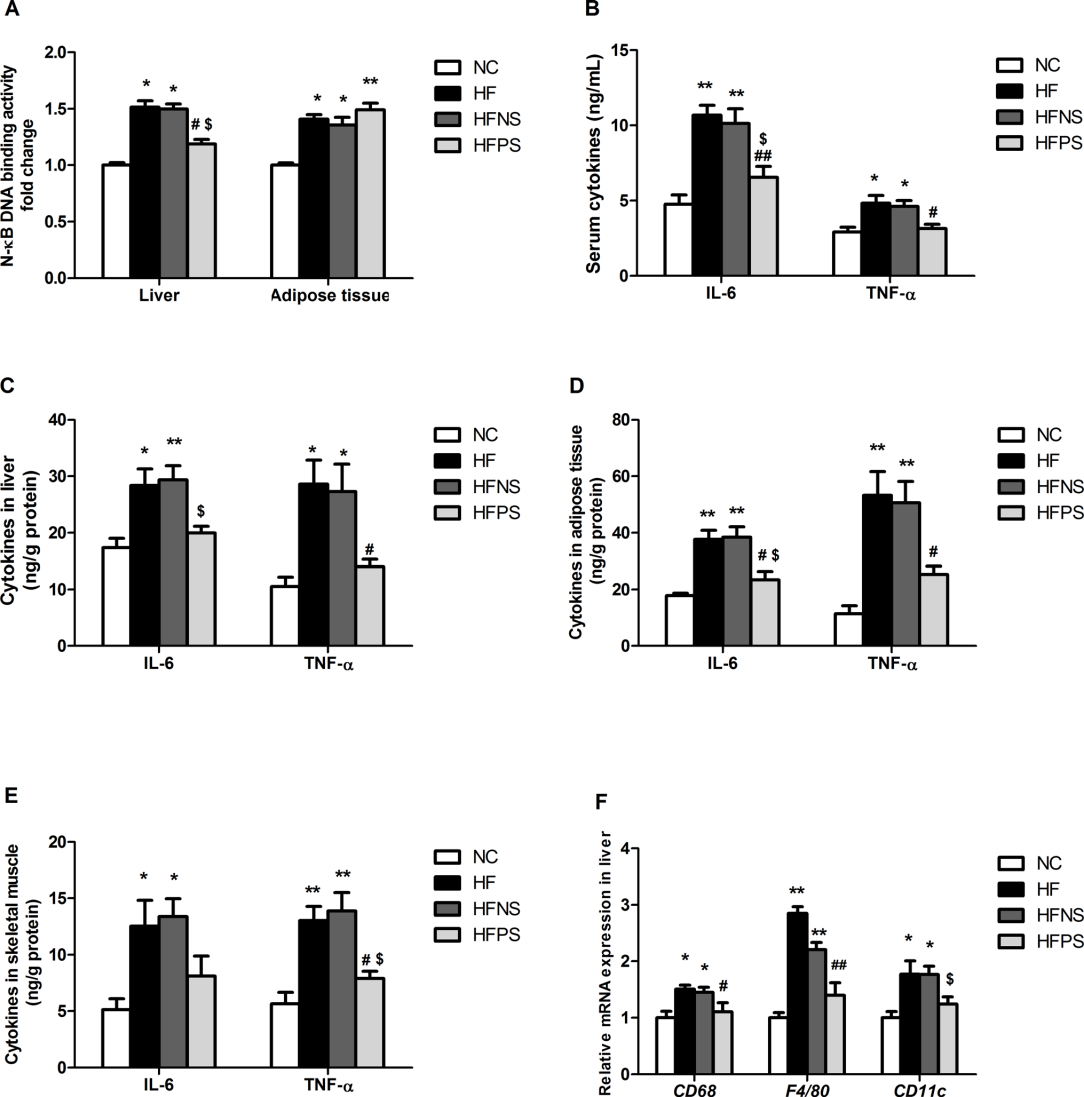
P65 siRNA administration inhibited inflammatory cytokines production in the circulation and tissues of HFD mice. (A) DNA binding activity of NF- kB (p65) was determined from liver and adipose tissue extracts by ELISA for each sample relative to the normal control. IL-6 and TNF-α levels were determined by ELISA in (B) Serum, (C) Liver, (D) Adipose tissue and (E) Skeletal muscle. (F) RT-qPCR of markers (*CD68*, *F4/80*, *CD11c*) of M1 macrophagesin liver. RT-PCR data are expressed as mean ± SE relative to NC values, arbitrarily set at 1. *p<0.05, **p<0.01 HF, HFNS and HFPS versus NC. ^#^p<0.05, ^##^p<0.01 HFPS versus HF. ^$^p<0.05, ^$ $^p<0.01, HFPS versus HFNS. n = 5–6. Data are shown as mean ± SE. NC (normal control group), HF (high-fat diet group), HFNS (high-fat diet with non-silence siRNA group) and HFPS (high-fat diet with P65 siRNA group).

It was observed that HF mice displayed enhanced NF-κB activation, parallel to HFNS mice’s level, in the hepatic and adipose tissue by 52% and 41%, respectively (Fig 1A). Interestingly, repeated P65 siRNA injection via tail vein of subject mice significantly inhibited NF-κB activation in liver but with little effect on adipose tissue. Meanwhile, high-fat diet (HFD) feeding elevated key pro-inflammatory cytokines, IL-6 and TNF-α, levels in circulation and tissues, including liver, epididymal fat, subcutaneous fat and gastrocnemius tissues (Fig 1B–1E). In agreement with the alteration of NF-κB, P65 siRNA administration decreased both inflammatory markers in serum and all the observed tissues.

In liver tissue, mRNA expression analyses of M1 macrophages immune activators including *CD68*, *F4/80* and *CD11c* were increased by approximately 50%, 180% and 80%, sequentially, in HF versus NC mice (Fig 1F). HFNS mice had a similar change with HF mice. When NF-κB was partially silenced, liver macrophages activation dropped significantly.

### P65 siRNA improved hepatic steatosis and insulin signaling in HFD feeding mice

**Fig 2:**
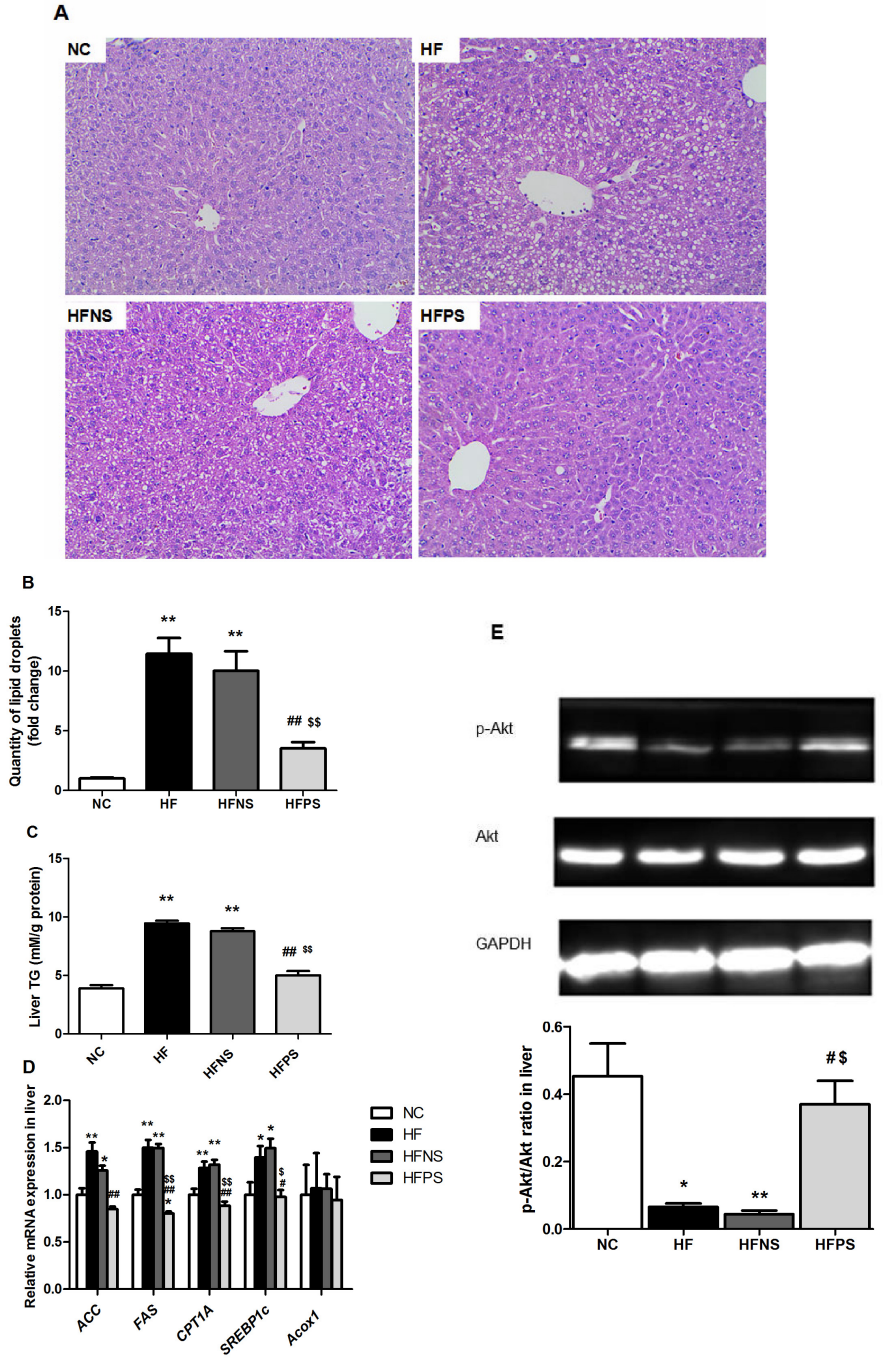
Improvement of hepatic steatosis and liver insulin signaling. (A) H&E staining of liver sections. Magnification: 200×. (B) Quantification of the degree of lipid droplets. (C) Liver triglyceride contents (n = 4–6). (D) Relative hepatic mRNA levels of genes related to lipid metabolism (n = 4–6). (E)Western blotting of insulin-stimulated Akt phosphorylation in liver. Representative western blot images and graphs representing the ratio of the (insulin-stimulated phospho-) protein of interest on Akt as measured by densitometry analysis are shown. *p<0.05, **p<0.01 HF, HFNS and HFPS versus NC. ^#^p<0.05, ^##^p<0.01 HFPS versus HF. ^$^p<0.05, ^$ $^p<0.01, HFPS versus HFNS. All data are presented as mean ± SE.

Histological analyses revealed that HF and HFNS mice had significantly more severe obesity-induced hepatic steatosis and lipid accumulation in terms of fat droplets volume and number (Fig 2A and 2B). Such changes were supported by increased liver triglyceride deposition in HF mice compared to NC mice (9.46±0.22 vs. 3.89±0.29 mmol/g protein, p<0.01, Fig 2C). Interestingly, P65 siRNA repeated injection pronounced reversed the hepatic lipid accumulation (p<0.01). Additionally, qPCR analyses of liver tissues showed that mRNA expression of genes involved in lipogenesis (*ACC*, *FAS*, *CPT1A and SREBP1c*) were attenuated by P65 siRNA treatment (Fig 2D). However, genes involved in fatty acid oxidation (*Acox1*) were unchanged (Fig 2D). Moreover, insulin-stimulated p-Akt in liver was decreased in HF and HFNS mice and markedly rebounded by P65 siRNA treatment (Fig 2E).

### P65 siRNA restored liver autophagy and enhanced activity of AMPK

**Fig 3:**
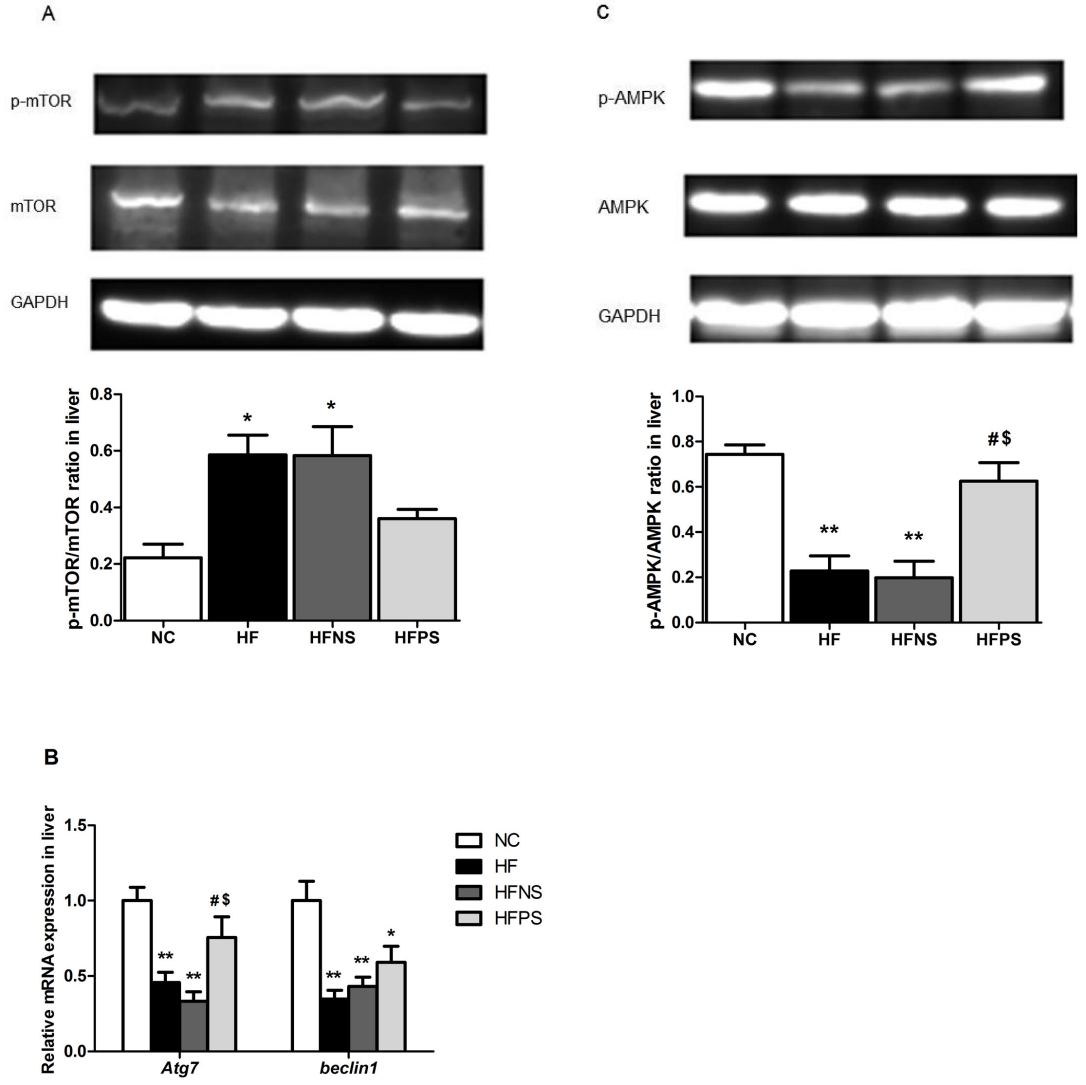
P65 siRNA enhances liver AMPK activation and increases the expression of key autophagy components. Western blotting of mTOR phosphorylation in liver (A), AMPK phosphorylation in liver (C) and RT-PCR of autophagy components (Atg7, Beclin1) in liver (B). Representative western blot images and graphs representing the ratio of the (insulin-stimulated phospho-) protein of interest on mTOR or AMPK as measured by densitometry analysis are shown. RT-qPCR data are expressed as mean ± SE relative to NC values, arbitrarily set at 1. *p<0.05, **p<0.01 HF, HFNS and HFPS versus NC. ^#^p<0.05, ^##^p<0.01 HFPS versus HF. ^$^p<0.05, ^$ $^p<0.01, HFPS versus HFNS. All data are presented as mean ± SE.

Autophagy is regulated by series of kinase, among them, mTOR is crucial. In our study, mTOR protein level in liver was significantly increased by about 3-fold on HF and HFNS mice compared to NC mice (Fig 3A), simultaneously, *Atg7* and *Beclin1* mRNA expressions were depressed to less than 50% (Fig 3B). As we know, the kinase mTOR is a negative regulator of autophagy induction, with activated mTOR suppressing autophagy, and inhibited mTOR promoting it [9]. And *Beclin1* and *Atg7* were necessary components of autophagosome formation. Hence the above markers expression of hepatic tissues for autophagy demonstrated a down-regulated autophagic flux, which was significantly reversed from both protein and transcript levels by P65 siRNA repeated injection. Interestingly, AMPK activation in liver tissue was markedly suppressed under chronic HFD feeding condition (Fig 3C). And this alteration was also significantly attenuated by P65 siRNA administration.

### P65 siRNA attenuated whole-body metabolic changes induced by chronic HFD feeding

**Fig 4:**
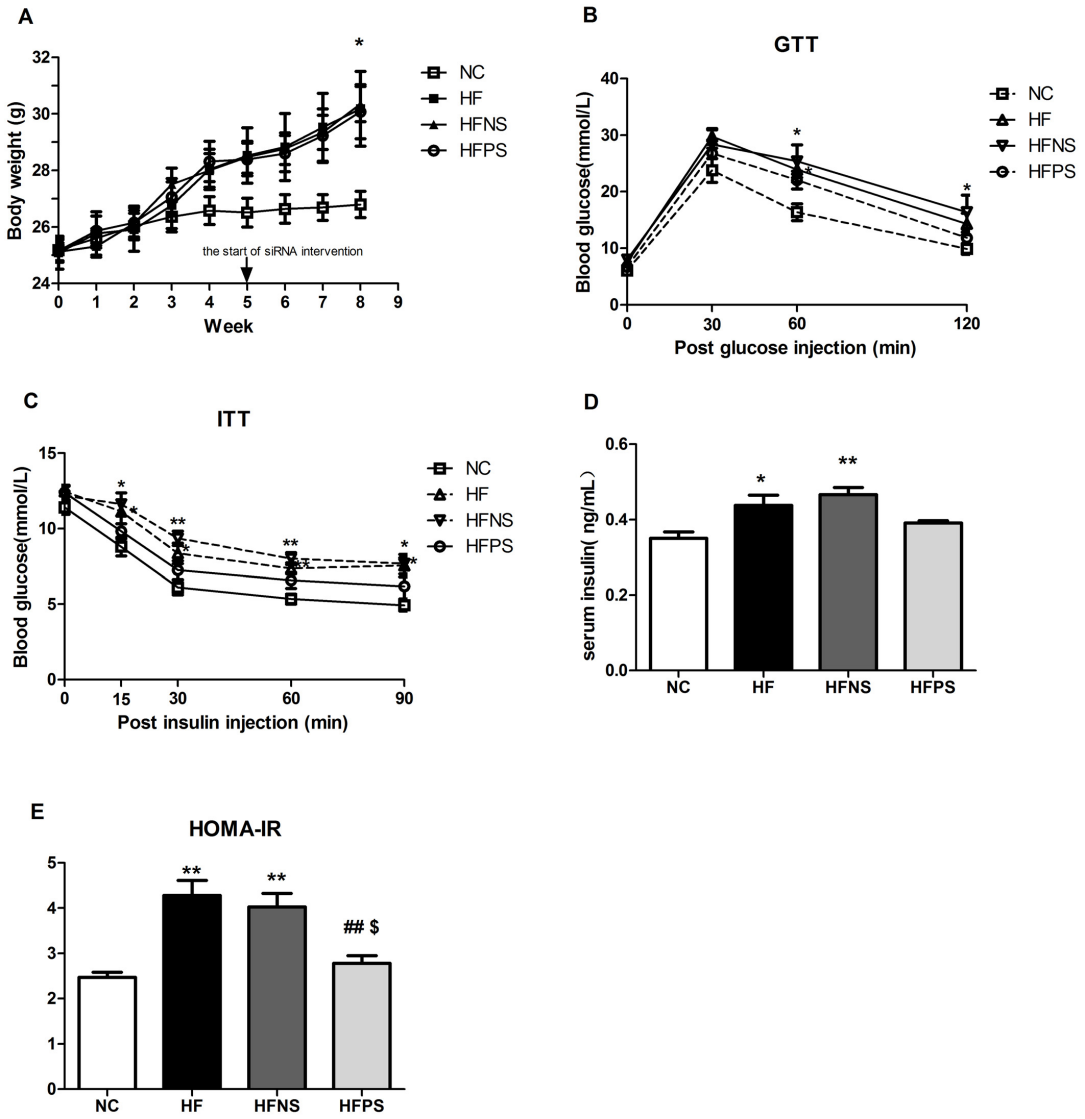
P65 siRNA partly protects mice from diet-induced systemic metabolic disorders. (A) Body weight curves of mice in different group over 8 weeks (n = 6–10). (B) Glucose tolerance test (GTT) in overnight fasted mice (n = 6–8). Glucose (2g/Kg BW) were injected intraperitoneally and tail vein blood samples were assessed for glucose concentration at the indicated time points (0min, 30min, 60min, 120min after injection). (C) Insulin tolerance test (ITT, 1U/Kg BW, n = 6–8) in mice fasted for 6h. Tail vein blood samples were assessed for glucose concentration at the indicated time points (0min, 15min, 30min, 60min, 90min after injection). (D) Serum insulin concentration in 6h fasted mice were measured by orbital blood samples (n = 5). (E) Insulin sensitivity was analyzed by HOMA-IR (n = 5). *p<0.05, **p<0.01 HF, HFNS and HFPS versus NC. ^#^p<0.05, ^##^p<0.01 HFPS versus HF. ^$^p<0.05, ^$ $^p<0.01, HFPS versus HFNS. All data are presented as mean ± SE.

At the end of 8 weeks, body weight (BW) in mice fed a high fat diet significantly outpaced mice fed normal chow diet (Fig 4A). There were no significant BW differences between HFD with or without P65 siRNA treatment mice (Fig 4A) and no differences in food intake were detected during the last four weeks (data were not shown). These results show that P65 siRNA treatment could not reverse diet-induced obesity.

HF and HFNS mice featured hyperlipidemia compared to normal diet mice, with serum FFA, TG and TC significantly elevated (Table 2). The FA contents of liver, epididymal fat, subcutaneous fat and gastrocnemius lysates were also analyzed. We observed higher FA concentrations in liver, fat and muscle tissues, all up to statistically significant except subcutaneous fat FA. Notably, mice with P65 siRNA administration were markedly protected from the diet-induced lipid disorders aforementioned. However, we did not assessment physical activity of the different group mice, but its affect was limited for single cage feeding and the same rearing environment.

**Table 2:** Lipid concentrations in blood and tissues. FFA, free fatty acids; TG, triglycerides; TC, total cholesterol; FA, fatty acid; mM/gprot,mmol/g protein.

	*Blood lipids(mmol/L)*			*Tissue lipids(mM/gprot)*			
	FFA	TG	TC	Liver FA	Epididymal FA	Subcutaneous FA	Gastrocnemius FA
**NC**	0.21±0.05	0.70±0.05	3.39±0.22	0.03±0.01	1.72±0.3	2.15±0.33	0.06±0.02
**HF**	0.69±0.12**	1.16±0.12**	4.38±0.2**	0.19±0.04**	6.57±1.13**	4.04±0.73	0.61±0.11*
**HFNS**	0.71±0.10**	1.32±0.11**	4.17+0.22*	0.12±0.02*	6.86±1.3**	4.24±0.19	0.53±0.11
**HFPS**	0.29±0.02^##^^$ $^	0.72±0.09^##^^$ $^	3.66±0.17^#^	0.05±0.01^##^^$^	2.89±0.5^#^^$^	2.55±0.46	0.26±0.05

*p<0.05

**p<0.01 HF, HFNS and HFPS versus NC.

^#^P<0.05

^##^P<0.01, HFPS versus HF.

^$^p<0.05

^$ $^p<0.01, HFPS versus HFNS. n = 4–6. All data are presented as mean ± SE.

Mice from different groups displayed similar glucose tolerance and insulin sensitivity before diet and siRNA treatment (S1 Fig). The glucose response during glucose tolerance test (GTT) was increased after HFD feeding for 4 weeks (S2 Fig), which even deteriorated during prolonged over nutrition (Fig 4B). Consistently, HF and HFNS mice become insulin insensitive and hyperinsulinemic (Fig 4C and 4D), while the impaired glucose and insulin tolerance were significantly improved in HFPS versus HF and HFNS mice. Moreover, HOMA-IR, a vital indicator of insulin resistance, was significantly increased by high-fat feeding (Fig 4E), which was partially down-regulated by P65 siRNA intervention.

## Discussion

Although evidence indicates that chronic low-grade inflammation state, now known as ‘‘metabolic inflammation”, is a critical contributing factor in the initiation and development of insulin resistance in obesity or caloric excess condition [22], the exact pathway(s) that transduce the inflammatory signal are obscure. Here we show that blockade of NF-κB mainly in liver, which is a key transcription factor involved in the regulation of gene expression in cytokines and pathways associated with inflammation, prevented HFD/obesity-induced hepatic steatosis, glucose intolerance, and abrogated insulin resistance in liver and systemic. Certainly, there must also be insulin resistance in adipose tissue for mounting studies have demonstrated it previously [23, 24]. What cannot be ignored is that the beneficial effects of P65 siRNA has little to do with body weight, because P65 siRNA administration did not reverse HFD related obesity in mice.

Interestingly, our results seem to reveal that P65 siRNA predominantly affected NF-κB transcriptional activity in liver other than adipose tissue, maybe due to chemically modified siRNA injected via tail vein generally easier to penetrate into the blood-rich organs like liver, heart, kidney and lung [25]. Moreover, Toll-like receptor 4 (Tlr4) is right the upstream of NF-κB signaling and, Jia et al.[1] have put it that Tlr4 activation on hepatocytes is responsible for obesity-related inflammation and insulin resistance. However, Tlr4 is highly expressed in macrophages and the causative role of macrophage-mediated inflammation in the pathogenesis of insulin resistance has also been well documented, so the proinflammatory activation of macrophages in liver, predominantly Kupffer cells, accounting for more than 80% of resident macrophages in the whole body, may also play a role. This has been confirmed by Neyrinck and Lanthier [26, 27] and is consistent with our results that gene expression of macrophages markers in liver, CD68, F4/80 and CD11c, were elevated in HFD feeding while significantly reversed by NF-κB silence, indicating that inhibition of macrophages in liver, maybe largely Kupffer cells, exerted ameliorating obesity and related metabolic disorders effects in HFD-fed mice. Taken together, it is possibility that inflammatory response in liver is amplified through crosstalk between activated Kupffer cells and impaired hepatocytes [1]. For the time being, this inter-cell action is just a hypothesis and ripe for further work in this field. Nevertheless, it is safe to draw a conclusion that liver is a critical source, not inferior to adipose tissue, of inflammation for metabolic disorders induced by obesity or over nutrition.

The above-mentioned could be concluded that caloric excess or diet-induced obesity triggered the activation of NF-κB pathway possibly via Tlr4 (of hepatocytes or Kupffer cells or both) resulting in the expression of proinflammatory cytokines such as TNF- α and IL-6, then amplification cascade of inflammation predisposed tissues and cells toward insulin resistance through the inhibition of insulin signaling. Hopefully, selective inhibition of NF-κB activity may be achieved by targeting specific tissue like liver to limit the side effects associated with global inhibitors of the NF-κB pathway.

Furthermore, our data showed that the improved insulin action in P65 siRNA treatment mice seemed to be in conjunction with enhanced liver autophagy. Autophagy deregulation during chronic caloric excess pushes the pathogenesis of multiple metabolic disorders [28–30]. Reports demonstrate autophagy restoration in liver can suppress “meta-inflammation” in obese mice [15]. There are also experimental evidence indicating that efficient autophagy activity can retard the activation of inflammasomes and induction of inflammatory responses [31, 32]. However, the possible crosstalk between NF-κB and autophagy signaling pathways is largely unknown. Our data demonstrated when NF-κB pathway was partly blocked by P65 siRNA, the activation of mTOR, known as an autophagy inhibitor, was decreased, though not reaching statistical significance. Therefore, mTOR may be one of the mediators between the two pathways, which has been proved by Djavaheri-Mergny et al [33]. Restored autophagy in liver, in turn, can facilitate lipid droplets degradation and thus improve fatty liver-associated pathologies such as hepatic steatosis and inflammation, ultimately leading to local and systemic insulin resistance. These studies provide evidence for a connection between inflammation signaling and autophagy and hint at the possibility of inducing selective autophagy to protect against HFD-induced insulin resistance.

Meanwhile, AMPK is reviewed as the major energy-sensor that activates a variety of catabolic processes and simultaneously inhibits several anabolic pathways involve in glucose and lipid metabolism and energy balance in multicellular organisms [34, 35]. A recent study demonstrated that upregulates activity of AMPK restores cardiac autophagic flux and ultimately enhances cardiac function in diabetic mice [36]. Hence, the ameliorated liver autophagy may also have something to do with AMPK activation in our study. Not unexpectedly, we observed a significantly reversed AMPK activity via P65 siRNA injections in our mice model of HFD-induced insulin resistance. Although the precise cellular mechanisms here require further clarification, the works done by Alers and Kim [34, 37] propose a possibility that AMPK and mTORC1 regulate autophagy through coordinated phosphorylation of Ulk1, a homologue of yeast Atg1.

However, controversy of the exact state of autophagy in the caloric excess situation exists: some publications report a suppressed autophagy [30, 38], whereas others claim a facilitated autophagic flux [39, 40] after a period of HFD feeding. This discrepancy might be explained by the fact that autophagy is a dynamic and highly regulated process impacted by diverse factors like species and ages of experimental animals, states of overfeeding, different methods used to examine autophagy as well as identification of basal and induced autophagy [41]. More importantly, it is challenging to discriminate the observed effects are just a secondary process or right caused by autophagy.

## Conclusion

In summary, we highlight that hepatic NF-κB pathway plays a pivotal role in diet-induced insulin resistance, and AMPK/mTOR-associated autophagy signaling seems to play a role in the above pathological process. Furthermore, silence of NF-κB using small interfering RNA partly abrogates features of HFD feeding mice, from inflammation, insulin resistance, hepatic steatosis to suppressed liver autophagic flux. This knowledge may facilitate novel therapeutic strategies to against diabetes associated with obesity and inflammation.

## Supporting Information

S1 Fig Basal glucose tolerance and insulin sensitivity after acclimatization. (A) Glucose tolerance test (GTT) in overnight fasted mice. Glucose (2g/Kg BW) were injected intraperitoneally and tail vein blood samples were assessed for glucose concentration at the indicated time points (n = 6–8) and corresponding area under the curve calculations for glucose values are shown (B). (C) Serum insulin concentration in 6h fasted mice were measured by orbital blood samples (n = 5). (D) Insulin tolerance test (ITT, 1U/Kg BW, n = 6–8) in mice fasted for 6h and corresponding area under the curve calculations for glucose values are shown (E). (F) Insulin sensitivity was analyzed by HOMA-IR (n = 5). *p<0.05, **p<0.01 HF, HFNS and HFPS versus NC. ^#^p<0.05, ^##^p<0.01 HFPS versus HF. ^$^p<0.05, ^$ $^p<0.01, HFPS versus HFNS. All data are presented as mean ± SE.
(TIF)

S2 Fig Glucose tolerance and insulin sensitivity after 4-week high-fat-diet feeding. (A) Glucose tolerance test (GTT) in overnight fasted mice. Glucose (2g/Kg BW) were injected intraperitoneally and tail vein blood samples were assessed for glucose concentration at the indicated time points (n = 6–8) and corresponding area under the curve calculations for glucose values are shown (B). (C) Serum insulin concentration in 6h fasted mice were measured by orbital blood samples (n = 5). (D) Insulin tolerance test (ITT, 1U/Kg BW, n = 6–8) in mice fasted for 6h and corresponding area under the curve calculations for glucose values are shown (E). (F) Insulin sensitivity was analyzed by HOMA-IR (n = 5). *p<0.05, **p<0.01 HF, HFNS and HFPS versus NC. ^#^p<0.05, ^##^p<0.01 HFPS versus HF. ^$^p<0.05, ^$ $^p<0.01, HFPS versus HFNS. All data are presented as mean ± SE.
(TIF)

## Abbreviations

NF-κB: nuclear factor-kappa B
HFD: high-fat-diet
NC: normal control group
HF: high-fat diet group
HFNS: high-fat diet with non-silence siRNA group
HFPS: high-fat diet with P65 siRNA group
p-AMPK: phosphorylation of AMP-activated protein kinase
p-mTOR: phosphorylation of mammalian target of rapamycin
ACC: acetyl CoA carboxylase
FAS: fatty acid synthase
CPT1A: carnitine palmitoyltransferase 1A
SREBP1c: sterol regulatory element binding protein-1c
Acox1: acyl-CoA oxidase 1
GTT: glucose tolerance test
ITT: insulin tolerance test
