# Supplementary figures and images for: Blocking Nuclear Factor-Kappa B Protects against Diet-Induced Hepatic Steatosis and Insulin Resistance in Mice
Source: PLoS One. 2016 Mar 1;11(3):e0149677. doi: 10.1371/journal.pone.0149677 (PMC4773172; doi:10.1371/journal.pone.0149677)

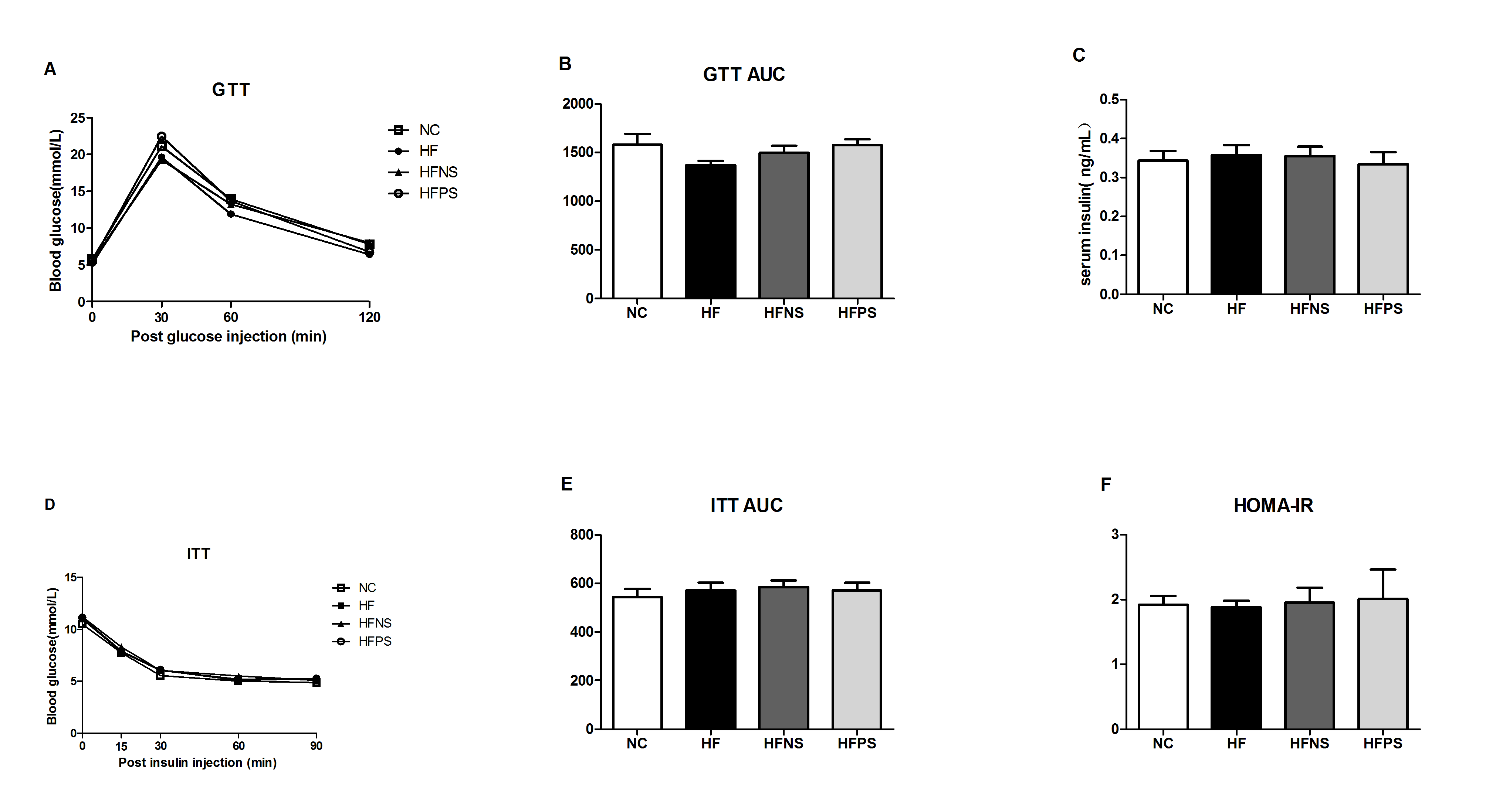

Supplement: S1 Fig — (A) Glucose tolerance test (GTT) in overnight fasted mice. Glucose (2g/Kg BW) were injected intraperitoneally and tail vein blood samples were assessed for glucose concentration at the indicated time points (n = 6–8) and corresponding area under the curve calculations for glucose values are shown (B). (C) Serum insulin concentration in 6h fasted mice were measured by orbital blood samples (n = 5). (D) Insulin tolerance test (ITT, 1U/Kg BW, n = 6–8) in mice fasted for 6h and corresponding area under the curve calculations for glucose values are shown (E). (F) Insulin sensitivity was analyzed by HOMA-IR (n = 5). *p<0.05, **p<0.01 HF, HFNS and HFPS versus NC. #p<0.05, ##p<0.01 HFPS versus HF. $p<0.05, $ $p<0.01, HFPS versus HFNS. All data are presented as mean ± SE. (TIF) [file pone.0149677.s001.tif]

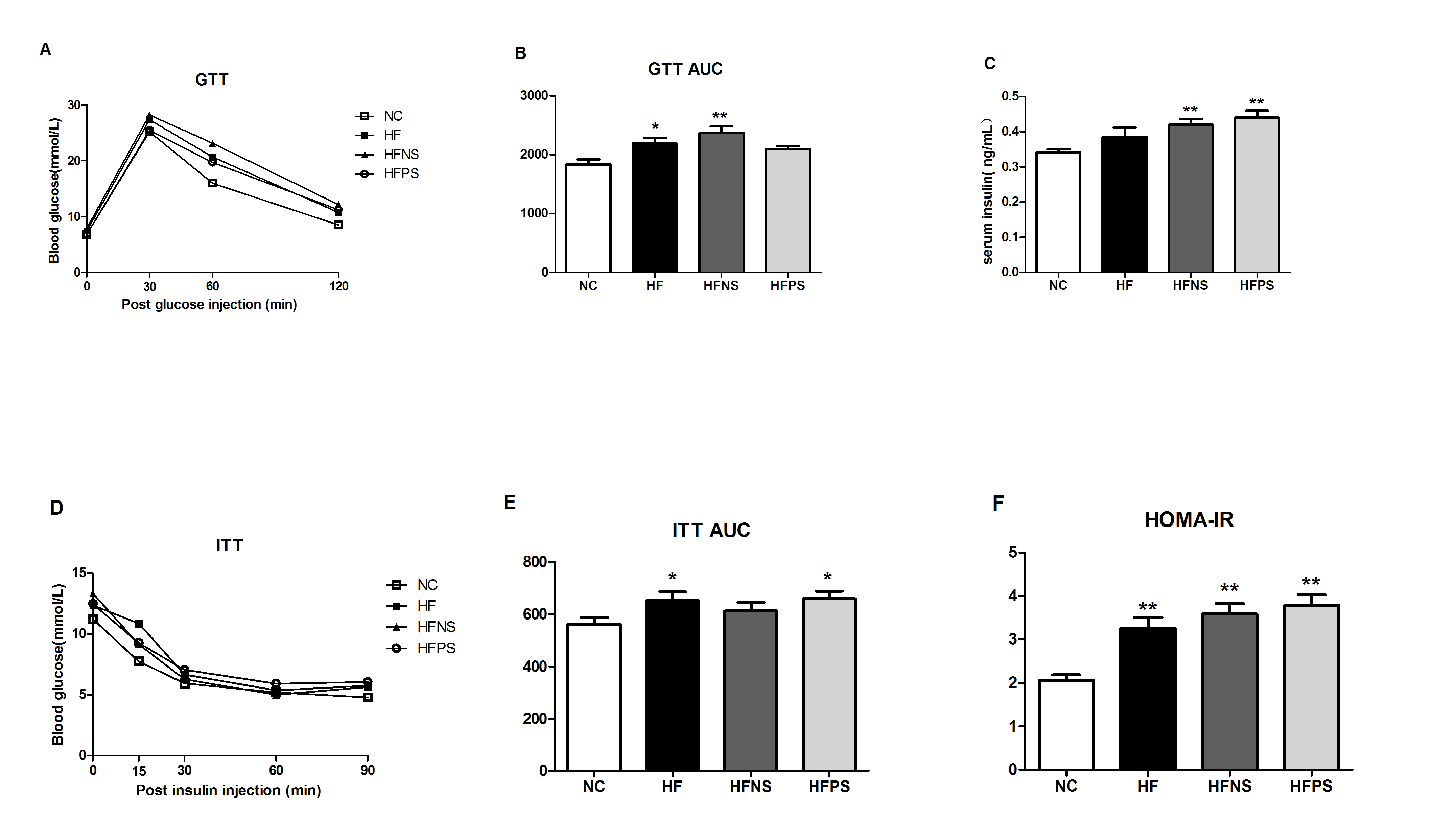

Supplement: S2 Fig — (A) Glucose tolerance test (GTT) in overnight fasted mice. Glucose (2g/Kg BW) were injected intraperitoneally and tail vein blood samples were assessed for glucose concentration at the indicated time points (n = 6–8) and corresponding area under the curve calculations for glucose values are shown (B). (C) Serum insulin concentration in 6h fasted mice were measured by orbital blood samples (n = 5). (D) Insulin tolerance test (ITT, 1U/Kg BW, n = 6–8) in mice fasted for 6h and corresponding area under the curve calculations for glucose values are shown (E). (F) Insulin sensitivity was analyzed by HOMA-IR (n = 5). *p<0.05, **p<0.01 HF, HFNS and HFPS versus NC. #p<0.05, ##p<0.01 HFPS versus HF. $p<0.05, $ $p<0.01, HFPS versus HFNS. All data are presented as mean ± SE. (TIF) [file pone.0149677.s002.tif]
